# IN TIME: ENTERAL NUTRITION FOR PRETERM INFANTS: SO MUCH LEARNED, BUT WE STILL DON’T KNOW HOW TO BEST FEED THEM

**DOI:** 10.1590/1984-0462/;2017;35;3;00018

**Published:** 2017-07-31

**Authors:** Jaques Belik

**Affiliations:** a Departamento de Pediatria e Fisiologia, University of Toronto. Hospital for Sick Children Toronto, Toronto, Ontário, Canadá.

Neonatology is a fast progressing science that revolutionized the care of preterm neonates. The past four decades provided us with the necessary technology and specific knowledge to prevent and treat respiratory distress syndrome, bacterial infection, hypoxic-ischemic encephalopathy and operable congenital anomalies in these infants. Interestingly, enteral nutrition support for the preterm neonate remains as a significant therapeutic challenge.

The newborn nutrition research has been mostly focused on addressing the requirements and the adequate balance of parenteral and enteral nutrition components, in order to promote postnatal growth that is similar to the intrauterine one.^1^ This has led to improved parenteral nutrition preparation, specialized preterm formulas, milk fortifiers and nutritional supplements that enable clinicians to currently treat the special needs of extremely premature infants.

Yet, one of the most common nutritional support challenges when it comes to caring for the preterm infant population is the presence of feeding intolerance.^2^ It is awkward to admit that the language commonly used amongst neonatal health care workers reflects our ignorance on the subject. A common interaction between a neonatal intensive care unit (NICU) nurse and the physician is best illustrated as follows.

- Nurse (often running after the physician holding a large syringe filled with some disgusting milk like fluid): Look at what I aspirated from that baby’’s stomach. What do you want me to do now?
- Physician: I do not know. How is the baby doing otherwise?
- Nurse: The baby looks fine and this is the first time today that I aspirated this much from her stomach.
- Physician: Well, just hold a feed and then resume at 50% of the feed volume to see what happens.

The reality is that most of the situations involving concerns about the feeding tolerance of the preterm infant relate to functional issues for which limited experimental data are available. The question that I often ask when faced with this situation is: What is going on? Is it the stomach that is not moving the milk forward, the pyloric sphincter that is not relaxing, or the issue is one of reduced bowel peristalsis?

Presently we lack the diagnostic means and sufficient knowledge about the preterm infant gastrointestinal physiology to adequately evaluate and manage these instances of feeding intolerance. Most of the studies addressing the factors responsible for gastric emptying in neonates were conducted over 30 years ago.^3^ ^,^ ^4^ ^,^ ^5^ ^,^ ^6^ ^,^ ^7^ ^,^ ^8^ Back in the 1980s, the preterm infants studied in these publications had an average gestational age of 30-34 weeks and were treated under different conditions than presently encountered in the modern NICU, thus limiting the applicability of their findings to the present era.

The therapeutic options presently available to enhance feeding tolerance are limited, mostly ineffective^9^ ^,^ ^10^ and likely detrimental.^11^ Methylxanthines, commonly used as respiratory stimulants, reduce gastric and intestinal motility,^12^ possibly contributing with the feeding intolerance of the preterm infant, given how widely this pharmacotherapy is used in this population.

Perhaps we, clinicians, ought to step back and adopt a more common sense approach to the nutritional support of preterm infants based on the term newborn feeding behavior. Normal term infants are introduced to the breast as soon as possible after birth, and allowed to nurse *ad lib.* Rodent data suggest that the newborn milk intake is regulated by stomach volume, and not satiety, as is the case later in life.^13^

Our present feeding guidelines for preterm infants are mostly based on the fear related to the occurrence of necrotizing enterocolitis (NEC), instead of on the physiologic needs of these neonates. Slow advancement of feeds are believed to prevent NEC, but there is limited, if any, reputable data to support this claim. Recently, many experts have recommended avoiding checking gastric aspirates to evaluate feeding tolerance, and there is data to support the fact that such strategy results in shorter time to reach full feeds.^14^

In summary, just as we researched and learned much about how to adequately provide for the nutritional needs of preterm infants, we need now to equally focus on the developmental aspects of their gastrointestinal function and regulation. We will have an answer for the nurse running after us with the syringe full of stomach content, but much research is required before we are ready for a proper answer.
